# GeneWaltz--A new method for reducing the false positives of gene finding

**DOI:** 10.1186/1756-0381-3-6

**Published:** 2010-09-28

**Authors:** Kazuharu Misawa, Reiko F Kikuno

**Affiliations:** 1 Research Program for Computational Science, Research and Development Group for Next-Generation Integrated Living Matter Simulation, Fusion of Data and Analysis Research and Development Team, RIKEN, 4-6-1 Shirokanedai, Minato-ku, Tokyo 108-8639, Japan; 2 Chiba Industry Advancement Center, 2-6 Nakase, Mihama-ku, Chiba 261-7126, Japan; 3 Kazusa DNA Research Institute, 2-6-7 Kazusa-Kamatari, Kisarazu, Chiba 292-0818, Japan

## Abstract

**Background:**

Identifying protein-coding regions in genomic sequences is an essential step in genome analysis. It is well known that the proportion of false positives among genes predicted by current methods is high, especially when the exons are short. These false positives are problematic because they waste time and resources of experimental studies.

**Methods:**

We developed GeneWaltz, a new filtering method that reduces the risk of false positives in gene finding. GeneWaltz utilizes a codon-to-codon substitution matrix that was constructed by comparing protein-coding regions from orthologous gene pairs between mouse and human genomes. Using this matrix, a scoring scheme was developed; it assigned higher scores to coding regions and lower scores to non-coding regions. The regions with high scores were considered candidate coding regions. One-dimensional Karlin-Altschul statistics was used to test the significance of the coding regions identified by GeneWaltz.

**Results:**

The proportion of false positives among genes predicted by GENSCAN and Twinscan were high, especially when the exons were short. GeneWaltz significantly reduced the ratio of false positives to all positives predicted by GENSCAN and Twinscan, especially when the exons were short.

**Conclusions:**

GeneWaltz will be helpful in experimental genomic studies. GeneWaltz binaries and the matrix are available online at http://en.sourceforge.jp/projects/genewaltz/.

## Introduction

The complete genome sequences of many organisms, including *Homo sapiens *[1,2] and *Mus musculus *[3], have been published. These studies have revealed that the majority of genes in the mammalian genome comprise non-coding regions and only a small percentage of genes comprise protein-coding regions. Thus, identifying protein-coding regions from nucleotide sequences is an essential step in genome analyses.

Thus far, a large number of computational methods have been developed for the prediction of protein-coding regions to facilitate gene identification studies [4-6]. Most gene prediction methods can be classified into 2 categories: *ab initio *methods and homology-based methods. The *ab initio *methods predict genes solely on the basis of signals of the target sequences and the model of gene structure [7,8]. Homology-based methods employ sequence similarity to known genes or proteins in the databases [9-11]. Most homology-based methods, such as Twinscan [12], incorporate the algorithms that are used in *ab initio *methods.

Wang et al. reported that the proportion of false positives among genes predicted by these methods are high, especially when the exons were short [13]. False positives among predicted GENSCAN result in waste of time and resources.

We developed GeneWaltz, a new filtering method for reducing the risk of false positives in gene finding. We focused on the fact that coding regions (CDSs) generally differ from non-coding regions by exhibiting a characteristic substitution pattern because of functional constraints on the protein sequences. For example, synonymous substitutions are more frequently observed in CDSs than nonsynonymous substitutions. GeneWaltz was named after the observation that the DNA sequence alignments of CDSs tend to have a single nucleotide difference after every 3 sites because of the synonymous substitutions that are frequently observed at the third positions of codons. By applying the theory of extreme value [14], GeneWaltz identifies candidate CDSs and tests whether these scores are significantly higher than those of the non-coding homologous sequences. Although GeneWaltz is a homology-based method, it does not use algorithms that are used in *ab initio *methods. GeneWaltz requires the comparison of 2 DNA sequences from different species but does not require any prior models of transcription, splicing, or translation.

## Algorithm

### Scoring scheme

GeneWaltz uses a lod score, which was first introduced by Dayhoff et al. [15], for measuring the similarity between 2 amino acids. To derive the lod score of an amino acid, the logarithm of the ratio of the observed frequency of a pair of amino acidsis divided by the random expected frequency of the same pair of amino acids. If the observed and expected frequencies are equal, the lod score is zero. A positive score indicates that a pair of amino acids is commonly observed, whereas a negative score indicates that a pair of amino acids is rarely observed. The general formula for any pair of amino acids is given as

(1) $$S_{ij}=\log\left(\frac{q_{ij}}{p_{i}p_{j}}\right),$$

where *S*_*ij *_is the score of the 2 amino acids *i *and *j*, *p_i _*and *p_j _*are their individual probabilities, and *q_ji _*is the frequency of the pair of amino acids *i *and *j*.

Because each codon has 3 nucleotides, we used the same scoring scheme for codon pairs

(2) $$S_{ijk,lmn}=\log\left(\frac{o_{ijk,lmn}}{e_{ijk,lmn}}\right),$$

where *S_ijk,lmn _*is the score of a codon pair *ijk *and *lnm*, and *i*, *j*, *k*, *l*, *m*, and *n *are nucleotides. This scoring scheme is similar to that of Zhang et al.[16]. *o_ijk,lmn _*and *e_ijk,lmn _*are the observed and expected frequencies of the codon pair *ijk *and *lnm*, respectively. A positive score indicates that a pair of codons is commonly observed in coding regions, while a negative score indicates that a pair of codons is rarely observed in coding regions.

In order to obtain the observed frequency in equation (2), we used the 7,645 orthologous gene pairs between the human and mouse genome as described by Clark et al. [17]. The alignment of these genes consists of 1,982,115 codons. We obtained the expected frequency of each codon pair from the alignment, and the expected frequency of each codon pair was the average of its observed frequency among the human-mouse orthologous codon pairs. Insertions, deletions, and undetermined sequences were excluded.

### Maximal Segment Pair

**Figure 1:**
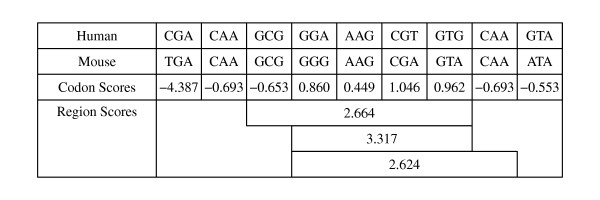
**Region Scores and Candidate Coding Regions (CDSs)**. The region score is the sum of the individual codon pair scores in an alignment. Human and mouse DNA alignment and codon scores are also shown. Three adjacent nucleotide pairs were treated as 1 codon pair. A high region score indicates that the region might be a CDS because that region contains many codon pairs with high scores. Note that the region scores should be calculated for all frames in both strands.

Let us define the region score as the sum of the individual codon pair scores in an alignment. An example of DNA sequence alignment between the human and mouse genome is shown in Figure 1. Three adjacent nucleotide pairs were treated as 1 codon pair, and the sum of the scores was calculated for all possible aligned regions. A high region score indicated many codon pairs with high scores in that region and suggested that the region was a coding region. GeneWaltz calculates the region scores for all frames in both strands.

Let us also define a Maximal segment pair (MSP) by the highest scoring pair of identical length segments chosen from 2 aligned sequences. The boundaries of an MSP are chosen to maximize its score; therefore, a MSP may be of any length. GeneWaltz heuristically attempts to calculate the MSP score, which provides a measure of the probability that any pair of sequences is within a protein-coding region. Our interest is in finding whole regions that are likely to be protein-coding regions. We, therefore, define a segment pair to be locally maximal if its score cannot be improved either by extending or by shortening both segments.

### Cutoff Value of the Score and Significance Level

It should be noted that although coding regions are expected to have high region scores, non-coding regions might have high region scores by chance alone. An important advantage of the MSP measure is that recent mathematical results allow the statistical significance of MSP scores to be estimated under an appropriate random sequence model [12,18].

For GeneWaltz, we developed a statistical test to examine whether the identified region was in actuality a coding region t by estimating the probability that those regions with a given region score appeared by chance. Karlin and Altschul [12] developed a theory of local alignment statistics for their BLAST search algorithm. Applying their theory, the probability (*P*) that a non-coding region with its length *N *has a MSP whose region score is greater than *S *by chance is approximately obtained by

(3) P=kNexp(−aS),

where *a *and *k *are constants. GeneWaltz can search all locally maximal segment pairs with scores above a specified cutoff. GeneWaltz tests the null hypothesis that the observed DNA sequence is not a protein-coding region by using equation (3).

We determined the values of *a *and *k *using computer simulations. Non-coding sequences were generated on the computer, and the GC content was set as 40% because the GC content of human and mouse genomes are approximately 40% [1-3]. Since the nucleotide identity between the human and mouse genome is approximately 70% [19], 30% of the nucleotides of the generated sequences were randomly selected and substituted by different nucleotides that were chosen to keep the average GC content the same. We generated 100 sequences of 100,000 bp. From these generated sequences, the regions with high scores were obtained by the algorithm described above, the number of high-scoring regions was counted, and the scores were recorded.

**Figure 2:**
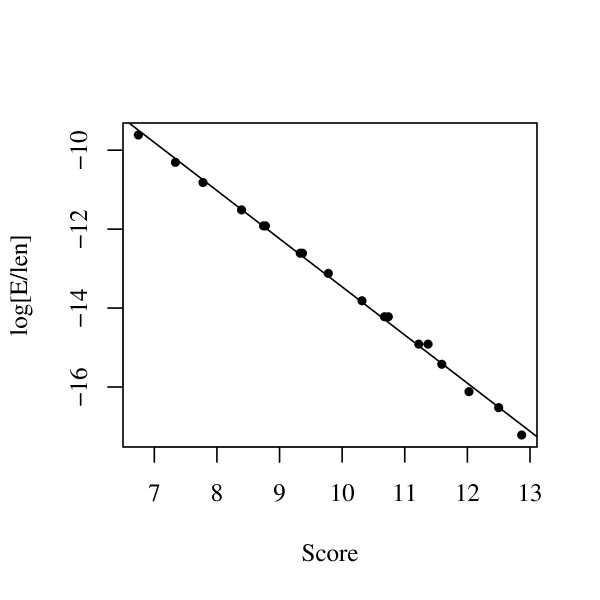
**Log-linear plot between the maximal segment pair (MSP) scores and their proportion of occurrences in the computer simulation**. The straight line is the regression line.

Figure 2 shows the scatter plot between the proportion of high-scoring regions and their scores. By using the log-linear regression method, *k *and *a *can be estimated by the least square method as *k *= 0.282 and *a *= 1.219. The regression line is shown in Figure 2.

## Performance Evaluation

### Materials

To evaluate the performance of gene prediction methods, we used the dataset referred to as Set 1 by Korf et al. [12]. The dataset was downloaded from the Twinscan website http://genes.cs.wustl.edu/. This dataset consists of 68 mouse genomic sequences and their top homologs from the human genome. The dataset was constructed by first searching the GenBank release 121 for all mouse sequences longer than 30 Kb that had annotated protein-coding regions. Pseudogenes were excluded from the data by searching for stop codons and frame shifts. The 68 mouse sequences comprised a total of 7.6 Mb with a mean length of 112 Kb and a median length 98 Kb. The data used to construct a codon substitution matrix shared some genes with this data set, but the proportion of overlapping genes was small.

### Methods

We utilized GENSCAN [7] and Twinscan 1.3 [12] to predict genes using the HumanIso.hmat as the parameter matrices and the Twinscan website http://genes.cs.wustl.edu/, respectively. For comparison, we divided the nucleotides into 2 categories: true positives and false positives. True positives were nucleotides of exons with a prediction that matched the annotation, whereas false positive regions were predicted as exons by the gene-finding method, but did not match the annotation. We assessed the performance of these methods by measuring the positive predictive value, the specificity, and the false positive rate. These values are defined as follows:

(4) $$\begin{array}{c} \text{The positive predictive valuse}=\frac{\text{true positives}}{\text{true positives}+\text{false positives}}\\ \text{The sensitivity}=\frac{\text{true positives}}{\text{true positives}+\text{false negatives}}\\ \text{The false positive rate}=\frac{\text{False positives}}{\text{true positives}+\text{false negatives}} \end{array}$$

In this paper, the predicted exons did not have to exactly match the true ones, and mismatch at the boundaries was accepted.

All predicted exons obtained by the gene-finding methods were tested by GeneWaltz by setting the cutoff value as P = 0.01. We conducted the chi-square test to compare the ratio of true positives to all positives to examine the effectiveness of GeneWaltz.

We evaluated gene-finding methods in terms of how successfully they identify true CDSs with few false positives, and summarized the results by plotting the partial receiver operating characteristic (partial ROC) curves by using various cutoff values. In order to obtain as many data points as possible, positives and negatives were counted based on the number of nucleotides instead of the number of exons when ROC curves were drawn.

## Results

The gene prediction results are shown in Table 1. Of the 3,061 exons predicted by GENSCAN, 1,818 exons were true positives and the rest were categorized as false positives. Of the 2,689 exons predicted by Twinscan, 2,209 exons were true positives and the rest were categorized as false positives.

**Table 1:** Numbers of True and False Positives in Gene Finding

	GENSCAN		Twinscan	
	
	True Positives	False Positives	True Positives	False Positives
Before GeneWaltz	1818	1243	2209	480
After GeneWaltz	1345	262*	1619	203*

*Significantly different.

When the exons predicted by GENSCAN were tested by GeneWaltz, 1,345 true positives passed the test but only 262 false positives passed the test (Table 1). When the exons predicted by Twinscan were tested by GeneWaltz, 1,619 true positives passed the test but only 203 false positives passed the test. The chi-square test showed that GeneWaltz significantly reduced the ratio of false positives to all positives predicted by both GENSCAN and Twinscan. The MHC genes did not pass the GeneWaltz test (data not shown).

**Figure 3:**
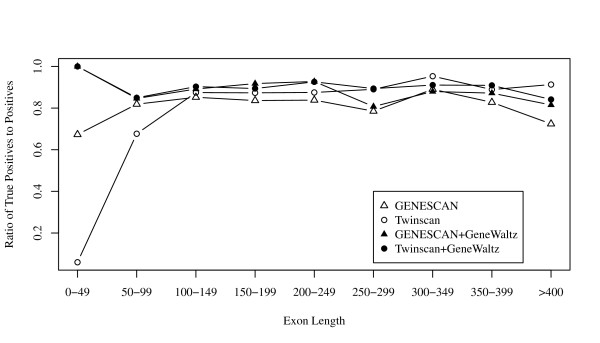
**Scatter plot of the ratios of true positives to all positives predicted by GENSCAN and Twinscan before and after filtering GeneWaltz versus exon length**. The unit of exon length is 3 nucleotides.

Figure 3 shows the relationship between the ratio of true positives to all positives obtained by gene-finding methods and the exon size. The exon size was measured by the number of codons, not by the number of nucleotides. This figure indicates that predicted genes contained a large number of false positives, especially when exon the length was shorter than 100 codons.

Figure 3 also shows that the positive predictive value was drastically improved by filtering these predicted genes by using GeneWaltz. The ratio of true positives to all positives and the exon length improved after filtering using GeneWaltz, especially when the exon lengths were short (Figure 3).

**Figure 4:**
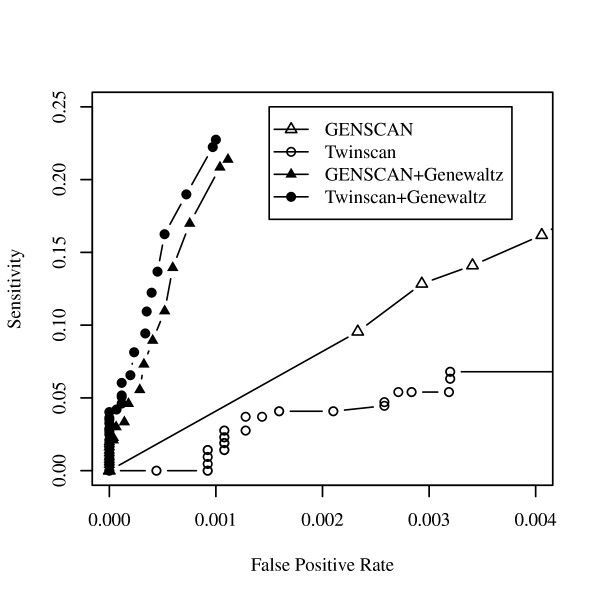
**The partial receiver operating characteristic (partial ROC) curves using Twinscan and GENSCAN across several GeneWaltz p-value thresholds**. A partial ROC curve plots the true positive rate for recovering true causal single-nucleotide polymorphisms (SNPs, *y*-axis) and the false positive rate (*x*-axis) over a range of small values of false positive rates.

Figure 4 shows the partial ROC curves using Twinscan and GENSCAN across several thresholds of the GeneWaltz P-value. A partial ROC curve plots the true positive rate for recovering true CDSs on the *y*-axis and the false positive rate on the *x*-axis over a range of small values of false positive rates.

## Discussion

We developed GeneWaltz, a new filtering method for testing coding regions. The ratio of true positives among all positives will be improved by the GeneWaltz filtering process, especially when the length of exon is longer than 100 codons.

There must be an open reading frame (ORF) in a region for a gene-finding method to predict a non-coding region as a gene. An ORF is a region between a start and a stop codon in the same frame, and such nucleotide triplets that do not actually code any amino acid sequences can occur by chance in genome sequences. However, ORFs that do not code amino acids are usually not very long, which is why a large portion of short predicted genes are false positives.

False positives in gene prediction indicate that our knowledge of coding regions is still limited. Studies to further elucidate gene structure information such as splicing sites, promoter regions, starting points for transcription and translation, will improve the accuracy of finding CDSs. However, DNA sequences do not always contain information about gene structures. For example, short sequences determined by next-generation sequencers [20] may not contain gene structure information. In such cases, GeneWaltz will be helpful for finding genes. The ROC curve showed that a high sensitivity was not achieved by GENESCAN and Twinscan by increasing the sensitivity of these methods by changing the program parameters. However, filtering using GeneWaltz yielded a high sensitivity.

For this evaluation, we constructed an empirical codon substitution matrix from orthologous gene pairs between mouse and human since we analyzed human genes. We are presently developing a general model of codon substitution [21] so that users can calculate a new scoring matrix using such codon substitution models in the future. GeneWaltz did not detect MHC genes, presumably because the matrix used in this study was an average of many genes whereas MHC genes have evolved under a positive selection pressure and show distinct nucleotide substitution patterns compared to other genes [22]. A specialized matrix might be necessary to detect such extraordinary proteins.

The current version of GeneWaltz are based on the sequence comparison of two species. If we can utilize the comparison of three or more genomes, better results will be obtained. Further studies of comparison of more genomes are required.

GeneWaltz binaries, the matrix, and the user manual are available at http://en.sourceforge.jp/projects/genewaltz/.

## Availability and requirements

• **Project name: **GeneWaltz

• **Project home page: **http://en.sourceforge.jp/projects/genewaltz/

• **Operating systems: **Platform independent

• **Programming language: **Java and C

• **Other requirements: **None

• **License: **MIT license

• **Any restrictions to use by non-academics: **License needed

## Competing interests

The authors declare that they have no competing interests.

## Authors' contributions

KM wrote the software and the manuscript. RFK supervised the project. Both authors read and approved the final manuscript.
